# Description of *Ortheziolamameti tranfagliai* new species (Hemiptera, Coccoidea, Ortheziidae) from India

**DOI:** 10.3897/zookeys.420.7890

**Published:** 2014-06-25

**Authors:** Éva Szita, Mehmet Bora Kaydan, Zsuzsanna Konczné Benedicty

**Affiliations:** 1 Plant Protection Institute, Centre for Agricultural Research, Hungarian Academy of Sciences, Herman Ottó u. 15, H-1022 Budapest, Hungary; 2 Çukurova Üniversity, Imamoglu Vocational School, Adana, Turkey

**Keywords:** Ensign scale, taxonomy, distribution, identification key, Oriental region

## Abstract

This paper describes a new *Ortheziolamameti* species from the Oriental region (India), namely *Ortheziolamameti tranfagliai* Konczné Benedicty, **sp. n.** The examined material was extracted from forest litter from India, using Berlese funnels. With this new species the genus *Ortheziolamameti* now includes six species. An identification key and distribution map are provided.

## Introduction

The family Ortheziidae (Hemiptera: Coccoidea), or ensign scale insects, have been considered to be one of the oldest families among the scale insects (Koteja 1996, Kozár and Miller 2000, Vea and Grimaldi 2012). According to Kozár (2004), the Ortheziidae family consists of four subfamilies: Ortheziinae Kozár, Newsteadiinae Kozár, Ortheziolinae Kozár and Nipponortheziinae Kozár. The subfamily Ortheziolinae is characterized by having: (i) the dorsum of the adult female entirely covered by wax plates, and a narrow band in midline of the dorsum, (ii) 3-segmented antennae (the size, shape, number, and type of setae highly variable), (iii) eye stalks protruding, thumb-like, fused with sclerotized area at base of antennae, sometimes called pseudobasal antennal segment, (iv) tarsi without digitules and (v) abdominal spiracles ventral on anterior segments, with at least one present on each side of abdominal segments I, II, or III; if present, posterior abdominal spiracles located on dorsum near anal ring surrounded by a cluster of multilocular disc pores. Ortheziolinae species are mainly found in the soil and distributed in the Palaearctic, Oriental and Ethiopian regions, and their host plant relationships are not well-known. In the subfamily there are four tribes, namely Ortheziolamametini Kozár, Ortheziolini Kozár, Matileortheziolini Kozár and Ortheziolacoccini Kozár.

Ortheziolamametini is characterized by having: (i) dorsum covered by wax plates, those in the middorsum being triangular, (ii) midthorax of venter without wax plates, and (iii) two spine bands inside the ovisac band. Species of Ortheziolamametini are distributed in the Ethiopian, Oriental and Palaearctic Regions. Although the genus is a typical member of the subfamily Ortherziolinae, it somewhat resembles *Arctorthezia* of subfamily Ortheziinae, known mainly in the northern part of the Holarctic region, in having the triangular wax plates on the middorsum. The genus includes 5 species, namely *Ortheziolamameti guineensis* (Morrison, 1954), *Ortheziolamameti loebli* (Richard, 1990), *Ortheziolamameti kosztarabi* (Kozár & Miller, 2000), *Ortheziolamameti maeharai* Tanaka & Amano, 2007, and *Ortheziolamameti taipensiana* Shia & Kozár, 2004; two of them are distributed in West Africa (*Ortheziolamameti guineensis* and *Ortheziolamameti kosztarabi*), two are in the Oriental Region (*Ortheziolamameti taipensiana* and *Ortheziolamameti loebli*), and one (*Ortheziolamameti maeharai*) is in the Far-East (Palaearctic).

In the present paper one new *Ortheziolamameti* species is described from the Oriental region (India). An identification key, distribution map and new additional locality records for the currently known *Ortheziolamameti* species are provided.

## Material and methods

The specimens described and recorded in this study were all collected using soil and litter sampling devices, and extracted by Berlese funnel. The samples are preserved in the Muséum d’Histoire Naturelle de Genève (MHNG) collection.

Specimens were prepared for light microscopy using the slide-mounting method discussed by Kosztarab and Kozár (1988). The morphological terminology used follows Kozár (2004), while the key was adopted from Kozár (2004) and Tanaka and Amano (2007).

The digital images of unmounted females were made with a Canon Eos400D camera and an MBC-10 stereomicroscope, and focus-stacking was processed by CombineZP software (Hadley 2010). All type material of the new species are deposited in the Muséum d’Histoire Naturelle de Genève (MHNG).

All measurements and counts were taken from all the material available and the values are given as a range for each character.

## Results and discussion

### Genus Ortheziolamameti

Taxon classificationAnimaliaHemipteraOrtheziidae

Kozár, 2004: 483.

#### Type species.

*Ortheziolamameti guineensis* Kozár, 2004, 484.

#### Diagnosis of genus.

**Figure 1.**
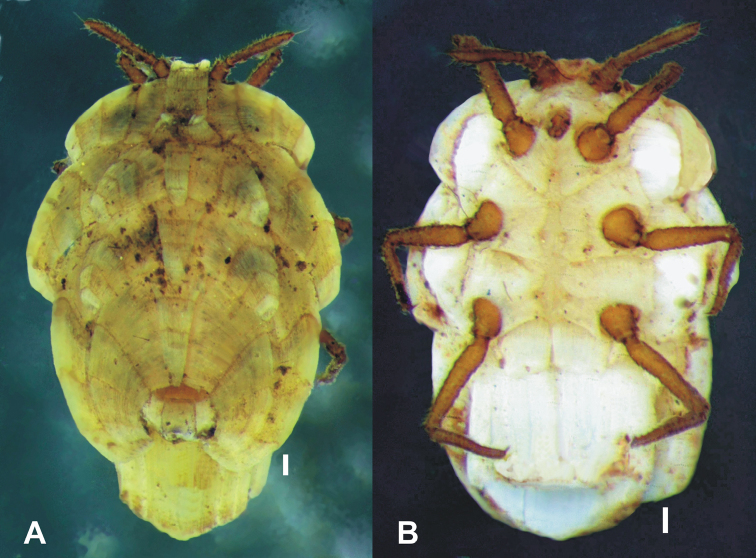
*Ortheziolamameti taipensiana* Shiau & Kozár, 2004, unmounted adult female **A** dorsal view **B** ventral view. Scale = 0.1 mm. Photo: É. Szita.

Dorsum of intact adult female covered with irregular and triangular shell-like wax plates; ventral thoracic wax plates around the appendages and on margin, thorax medially lacking wax plates; wax cover corresponding to wax plates on slide-mounted specimens on both sides (Kozár 2004) (Fig. 1).

Slide-mounted adult female with 3-segmented antenna; third antennal segment with slender apical seta, flagellate sensory seta and small subapical seta; second segment with 1 sensory pore. Eye stalk protruding, thumb-like, fused with base of antenna. Legs well developed; leg setae robust, spine-like; trochanter and femur fused, tibia and tarsus fused; tibia with 1 sensory pore and at least 1 fleshy sensory seta; tarsus without tarsal digitules; claw digitules hair-like, claw without a denticle. Labium 1 segmented, with many setae; with 3 long setae near apex of labium, setae very close together, all situated in a single setal socket. Anal ring situated in a fold of derm on dorsal surface, ring bearing 6 setae. Sclerotized plate present on dorsum anterior to anal ring, wider than long. Thumb-like pores forming a cluster on each side of anal ring. Abdominal spiracles in centre of multilocular disc pore cluster present laterad to anal ring (Kozár 2004).

*Distribution*. The six *Ortheziolamameti* species are distributed in the Ethiopian, Oriental and Palaearctic regions (Fig. 3). For detailed distribution data of previously known five species, see ScaleNet (Ben-Dov et al. 2013). New locality records for several *Ortheziolamameti* species were discovered during the study of the MHNG collection, which are listed below.

#### Key to species of *Ortheziolamameti*

**Table:** 

1	Setae on antennae hair-like	2
–	Setae on antennae spine-like	5
2	Multilocular disc pores absent around vulva	3
–	Multilocular disc pores present around vulva	4
3	Plates 13, 17 and 18 resembling very small groups of spines, without cluster of spines between hind legs, second spine band in ovisac area in a sparse row	*Ortheziolamameti tranfagliai* sp. n.
–	Plates 13, 17 and 18 in complete groups of spines, large cluster of spines between hind legs with a second spine band in ovisac area forming a complete row	*Ortheziolamameti loebli*
4	Wax plate 9 narrow anteriorly, so that wax plate 9 is shaped like an elongated isosceles triangle; wax plate 10 similar in shape to wax plate 9, not so wide anteriorly	*Ortheziolamameti taipensiana* Shiau & Kozar
–	Wax plate 9 wide anteriorly, so that wax plate 9 almost forms an equilatertal triangle; wax plate 10 not similar to wax plate 9, but norrow posteriorly and widening anteriorly	*Ortheziolamameti maeharai* Tanaka & Amano
5	Multilocular disc pores present in a band anterior of each spine band within ovisac	*Ortheziolamameti kosztarabi*
–	Multilocular disc pores present in a row only in anterior spine band within ovisac	*Ortheziolamameti guineensis*

### Ortheziolamameti tranfagliai

Taxon classificationAnimaliaHemipteraOrtheziidae

Konczné Benedicty sp. n.

http://zoobank.org/309E49AD-10F9-4EF6-B764-04985114D053

Figure 1

#### Material examined.

*Holotype*. Adult female. Slide with two specimens, holotype clearly marked, signed red. India, Kerala, Cardamon Hills, 26.12.1972, Leg. Bes/Löbl [MHNG code: Bes/Löbl (50), PPI code: 9807].

*Paratypes*. 3 adult females, 1 specimen on same slide as holotype, 2 specimens on a separate slide, same data as holotype.

#### Description.

*Unmounted female*. Not seen.

**Figure 2.**
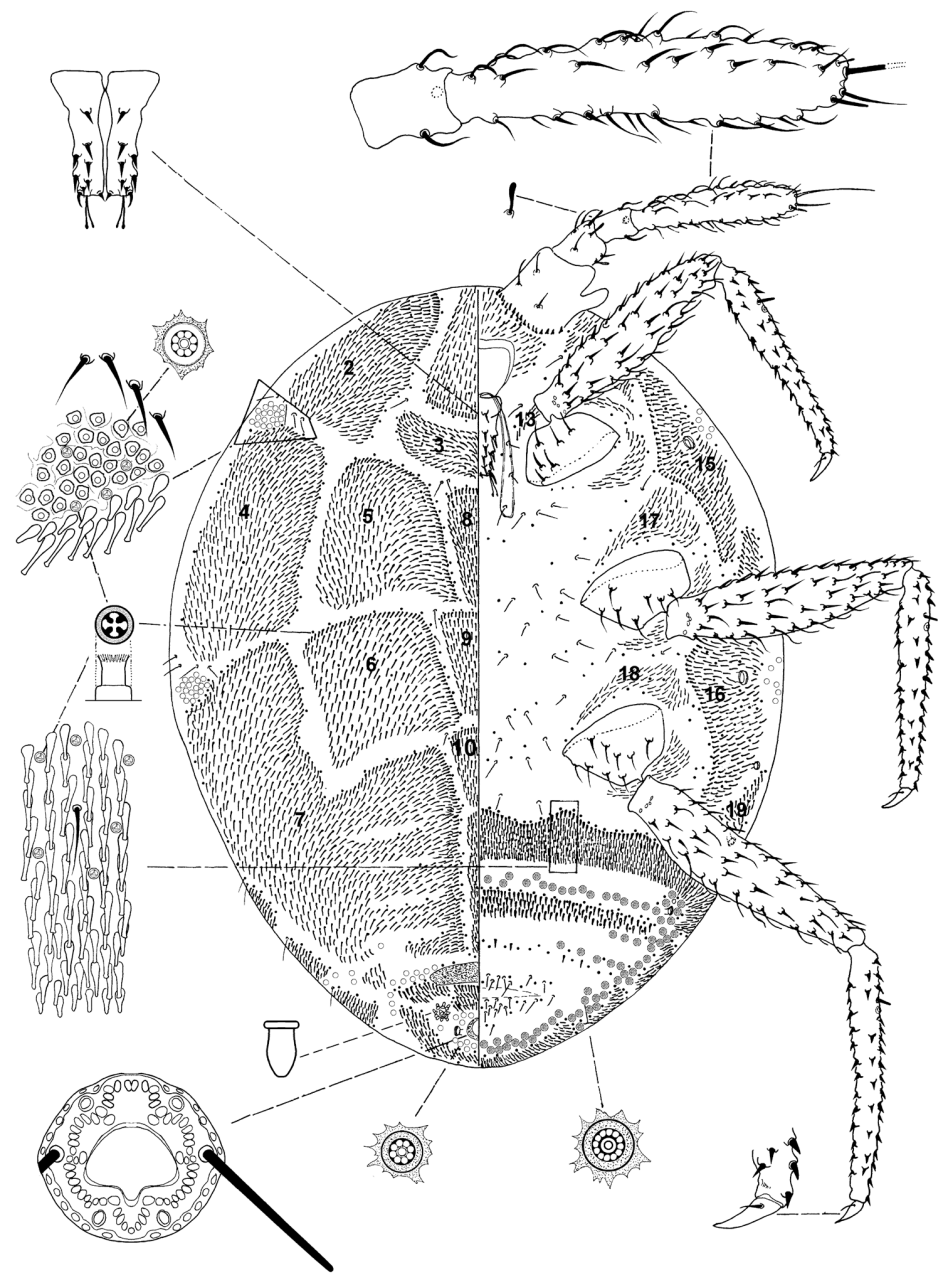
*Ortheziolamameti tranfagliai* Konczné Benedicty sp. n., holotype, mounted adult female.

*Mounted female* (Figure 2).

*Adult female*. 1.554–1.709 mm long; 1.114–1.295 mm wide. Length of antennal segments: 1^st^ 72–101 µm, 2^nd^ 60–76 µm; 3^rd^ 290–372 µm; 3^rd^ segment nearly parallel sided; apical seta of antenna 146–180 µm long; subapical setae 55–61 µm long; flagellate sensory setae near apical seta 24–30 µm long; microsetae absent from apex of antenna; without unusual hair-like setae near subapical setae; with several small setae near posterior edge of antennae; all segments of antennae covered with many hair-like, curved, pointed setae, longest seta 25 µm long; first antennal segment with 2 capitate sensory setae on each side; third segment with 27–37 setae.

*Venter*. Labium 151–168 µm long. Stylet loop slightly longer than labium. Leg segment lengths: front coxa 101–144 µm, middle 120–156 µm, hind 144–178 µm; front trochanterfemur 312–382 µm, middle 331–398 µm, hind 357–434 µm; front tibia-tarsus 323–382 µm long, middle 357–408 µm, hind 434–525 µm; front claw 45–60 µm, middle 48–55 µm, hind 53–60 µm; claw digitules 17–21 µm long, legs with longitudinal rows of robust setae, longest seta on trochanter-femur 16 µm; with 1 or 2 flagellate sensory seta on tibia-tarsus each 22 µm long; trochanter with 3 or 4 sensory pores on each surface. Wax plates present on marginal areas of head and thorax, with wide marginal wax band laterad of each thoracic spiracle (plates 15 and 16); plates 13, 17 and 18 resembling very small groups of spines; without a cluster of spines between hind legs; with 2 bands of spines within ovisac band, second one with an incomplete row of spines. Thoracic spiracles usually associated with 3 or 4 multilocular disc pores, each pore with 8 loculi, 6 µm in diameter; diameter of anterior thoracic spiracles 20–26 µm. Flagellate setae few, with several setae near anterior edge of ovisac band, with several setae associated with anterior and posterior multilocular disc pore rows.

Quadrilocular pores 3 µm in diameter, scattered in the ovisac band. Multilocular disc pores with 8–12 loculi around perimeter, one loculus in central hub; 7–8 µm in diameter; present near anterior edges of spine bands, scattered along lateral edge of each ovisac band. Abdominal spiracles present anterior of ovisac band; without sclerotized vestibule.

*Dorsum*. Wax plates covering the whole surface; mediolateral thoracic plates large (plates 3, 5 and 6), covering most of mediolateral thoracic areas; medial area without wax plates, this area with 3 triangular wax plates (plates 8, 9 and 10). Spines at margin of wax plate 4, each 12–14 µm long, in middle of wax plates 18–21 µm long; spines apically capitate. Setae present in marginal clusters near posterior edges of marginal wax plates (plates 2 and 4), with 3 or 4 setae laterad of each thoracic spiracle, 27–30 µm long; also present in very small numbers on other wax plates. Quadrilocular pores 3 µm in diameter, scattered, mainly along margins of wax plates. Multilocular disc pores present in a cluster near anal ring, near the sclerotized plate, and on the margin at the level of ventral thoracic spiracles. Sclerotized plate 80–120 µm long, 265–330 µm wide. Anal ring with two complete rows of round pores (4–5 µm in diameter); three pairs of anal ring setae each 45–21 µm long; anal ring 43–53 µm wide, 43–53 µm long. Thumb-like pores forming a cluster on each side of anal ring, each 5–8 µm long. Modified pores (quadrilocular pores) 3–4 µm long, scattered on surface. The abdominal spiracle is located in centre of the multilocular disc pore clusters laterad to anal ring.

#### Ecology.

*Host plants*. Unknown. Collected from forest litter.

#### Distribution.

India.

**Figure 3.**
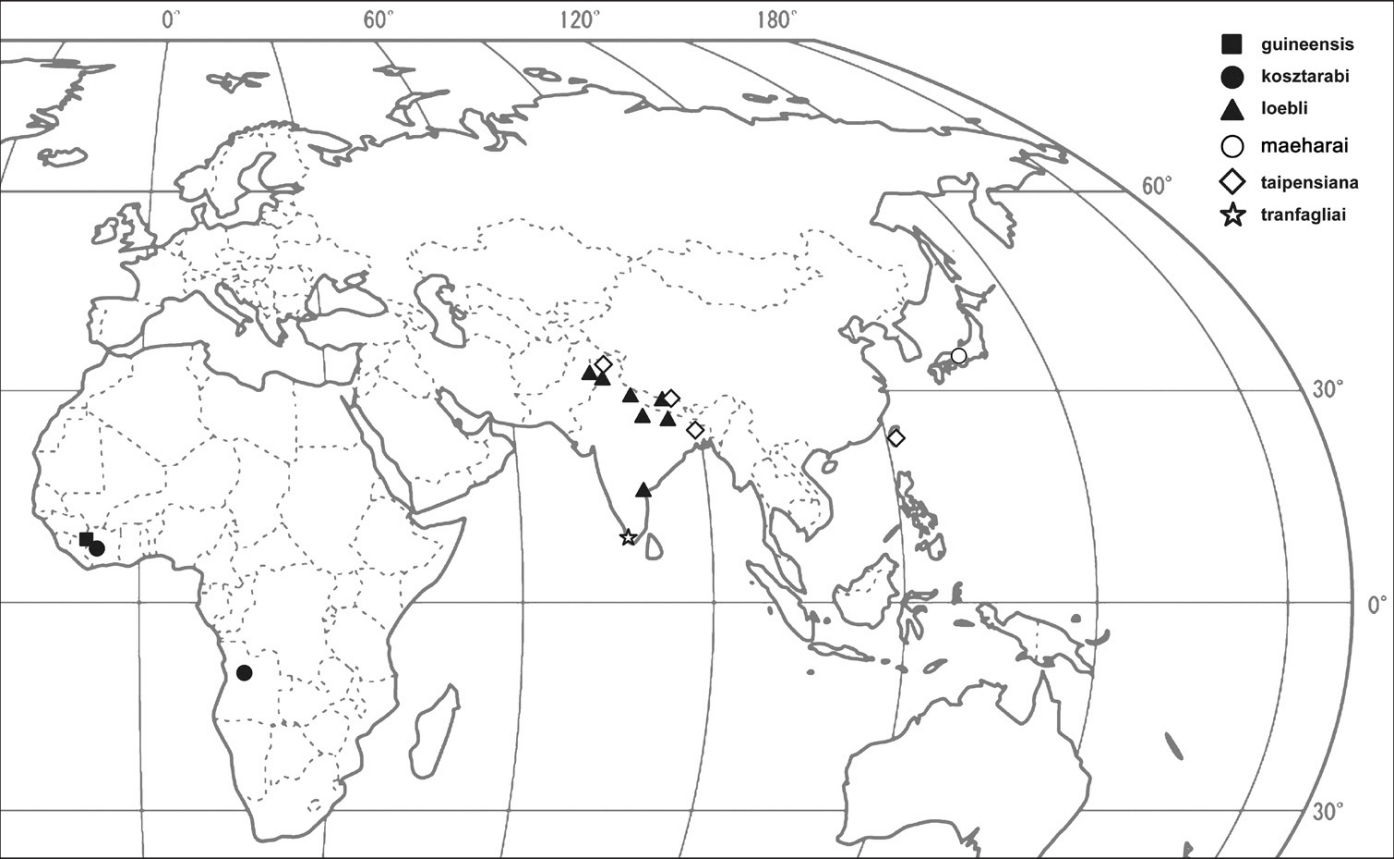
Distribution map of *Ortheziolamameti* species in the world.

#### Etymology.

This species is named after the Italian coccidologist Dr. Antonio Tranfaglia.

#### Comments.

*Ortheziolamameti tranfagliai* sp. n. can be recognized by the following combination of characters: (i) having hair-like setae on antennal segments, (ii) having two spine bands in the ovisac area and (iii) lacking multilocular disc pores around vulva. *Ortheziolamameti tranfagliai* is similar to *Ortheziolamameti loebli* in having hair-like setae on antennae and lacking multilocular disc pores around vulva, but differs from *Ortheziolamameti loebli* in the following characters (those of *Ortheziolamameti loebli* in brackets), (i) plates 13, 17 and 18 resembling very small groups of spines (plates 13, 17 and 18 complete); (ii) without cluster of spines between hind legs (with large cluster of spines between hind legs) and (iii) second spine band in ovisac area in a sparse row (in a complete row).

### Distribution of *Ortheziolamameti* species in the world

***Ortheziolamameti guineensis* (Morrison, 1954)**

Distribution. Guinea. Distribution note of Ghana (Ben Dov et al. 2013, Kozár 2004) might be a mistranslation of French Guinea mentioned by Morrison (1954). The Nimba Mountains where the type species originates from, currently a national park in Guinea. New records: Ivory Coast, Man, Cascades, 7.10.1980, leg. V. Mahnert, L. Perret [MHNG code: 80/12 Cote d’Ivorie, PPI code: 9624].

***Ortheziolamameti kosztarabi* (Kozár & Miller, 2000)**

Distribution. Angola (Kozár and Miller 2000).

***Ortheziolamameti maeharai* Tanaka & Amano, 2007**

Distribution. Japan (Tanaka and Amano 2007).

***Ortheziolamameti loebli* (Richard, 1990)**

Distribution. Nepal (Richard 1990; Ben-Dov et al. 2013). New records: India, Khajjiar, East of Dalhousia, 21.10.1988, leg. S. Vit [MHNG code: 30.INDE, PPI code: 9646]; India, Uttar Pradesh, Garhwal, 27.10.1979, leg. I. Löbl [MHNG code: Löbl (26), PPI code: 9803]; India, Uttar Pradesh, Kumaon, Rangarh, 9.10.1979, leg. I. Löbl [MHNG code: Löbl (6, 7), PPI code: 9789, 9816]; India, Uttar Pradesh, Kumaon, Chambattiva prés Ranikhet, 12–13.10.1978, leg. I. Löbl [MHNG code: Löbl (10), PPI code: 9811]; India, Madras, Anai matai Hills, 18.11.1972, leg. Besuchet, Löbl [MHNG code: Bes/Löbl (35), PPI code: 9815]; India, Madras, Nilgiri, 22.11.1972, leg. I. Löbl [MHNG code: Löbl (22), PPI code: 9809]; Nepal, Bagmati, Bahrabise, North-East of Dobate Ridge, 2700 m a.s.l., 2.05.1981, leg. Löbl, A. Smetana [MHNG code: Löbl, A. Smetana (54), PPI code: 8930]; Nepal, Bagmati, Malemchi, 2900 m a.s.l., 14.04.1981, leg. Löbl, Smetana [MHNG code: 12. INDE, PPI code: 9649]; Pakistan, Punjab, Murree, 1950 m a.s.l., 23.04.1984, leg. S. Vit [MHNG code: Pak-84/22, PPI code: 8948].

***Ortheziolamameti taipensiana* Shiau & Kozár, 2004**

Distribution. Taiwan, Thailand (Kozár 2004). New records: India, Tigerhill, 25002600 m a.s.l., 18.10.1978, leg. Besuchet, Löbl [MHNG code: Besuchet/Löbl (19), PPI code: 9631]; India, Meghalaya, Khasi Hills, 1850 m a.s.l., 28.10.1978, leg. Besuchet, Löbl [MHNG code: Besuchet/Löbl (27), PPI code: 9638]; Nepal, Bagmati, Malemchi, 2900 m a.s.l., 14.04.1981, leg. Löbl, Smetana [MHNG code: Löbl/ Smetana (23), PPI code: 9648].

## Supplementary Material

XML Treatment for Ortheziolamameti

XML Treatment for Ortheziolamameti tranfagliai
